# Using a quantitative quadruple immunofluorescent assay to diagnose isolated mitochondrial Complex I deficiency

**DOI:** 10.1038/s41598-017-14623-2

**Published:** 2017-11-15

**Authors:** Syeda T. Ahmed, Charlotte L. Alston, Sila Hopton, Langping He, Iain P. Hargreaves, Gavin Falkous, Monika Oláhová, Robert McFarland, Doug M. Turnbull, Mariana C. Rocha, Robert W. Taylor

**Affiliations:** 1 Wellcome Centre for Mitochondrial Research, Institute of Neuroscience, Newcastle University, Newcastle Upon Tyne, UK; 2 NHS Highly Specialised Mitochondrial Diagnostic Laboratory, Newcastle upon Tyne Hospitals NHS Foundation Trust, Newcastle upon Tyne, UK; 3 The National Hospital for Neurology and Neurosurgery, University College London Hospitals NHS Foundation Trust, London, UK; 4 Present Address: School of Pharmacy and Biomolecular Sciences, Liverpool John Moores University, Byrom Street, Liverpool, L3 3AF UK; 5 Present Address: BHF Centre of Research Excellence, The James Black Centre, King’s College London, University of London, 125 Coldharbour Lane, London, SE5 9NU UK

## Abstract

Isolated Complex I (CI) deficiency is the most commonly observed mitochondrial respiratory chain biochemical defect, affecting the largest OXPHOS component. CI is genetically heterogeneous; pathogenic variants affect one of 38 nuclear-encoded subunits, 7 mitochondrial DNA (mtDNA)-encoded subunits or 14 known CI assembly factors. The laboratory diagnosis relies on the spectrophotometric assay of enzyme activity in mitochondrially-enriched tissue homogenates, requiring at least 50 mg skeletal muscle, as there is no reliable histochemical method for assessing CI activity directly in tissue cryosections. We have assessed a validated quadruple immunofluorescent OXPHOS (IHC) assay to detect CI deficiency in the diagnostic setting, using 10 µm transverse muscle sections from 25 patients with genetically-proven pathogenic CI variants. We observed loss of NDUFB8 immunoreactivity in all patients with mutations affecting nuclear-encoding structural subunits and assembly factors, whilst only 3 of the 10 patients with mutations affecting mtDNA-encoded structural subunits showed loss of NDUFB8, confirmed by BN-PAGE analysis of CI assembly and IHC using an alternative, commercially-available CI (NDUFS3) antibody. The IHC assay has clear diagnostic potential to identify patients with a CI defect of Mendelian origins, whilst highlighting the necessity of complete mitochondrial genome sequencing in the diagnostic work-up of patients with suspected mitochondrial disease.

## Introduction

Mitochondrial diseases are a group of clinically heterogeneous disorders caused by dysfunction of the oxidative phosphorylation (OXPHOS) system. Accounting for approximately 30% of all cases, isolated Complex I (CI) deficiency is the most frequently observed biochemical manifestation of an OXPHOS defect in patients, particularly within a paediatric setting^1^. With a relative molecular mass of 1MDa, mitochondrial CI (NADH: ubiquinone oxidoreductase) is the largest complex of the respiratory chain, oxidising NADH to NAD^+^ and playing a central role in both electron transfer and cellular energy (ATP) production. The complex is L-shaped in structure, consisting of 2 fragments; a hydrophilic peripheral arm, which extends into the matrix of the mitochondria and a hydrophobic arm which is embedded into the inner mitochondrial membrane. The complex is comprised of 45 subunits, 7 of which are encoded by the mitochondrial DNA (mtDNA) and the remaining 38 subunits encoded by the nuclear DNA (nDNA)^2^. This structure can be further divided into 3 functional modules: within the peripheral arm the N module (which binds and oxidizes NADH, providing electrons to the Fe-S clusters) and the Q module (where ubiquinone is reduced to ubiquinol) whilst the membrane arm contains the third module known as the P module which catalyses proton transfer^3–8^.

The assembly of CI is a multistep process; recent studies propose a modular assembly model, in which five assembly intermediates - termed Q/Pp-a, Pp-b, PD-a, PD-b and N - form separately before assembling into higher molecular mass intermediates to subsequently form the completed holoenzyme^5^. Despite differences in the nomenclature of assembly intermediates, all proposed models described in the recent literature follow the same sequence of steps^9–12^. Briefly, the first step involves formation of the Q module, followed by the addition of the mitochondrial encoded ND1 core subunit (early stage of assembly). Next, the membrane arm is assembled, starting with the insertion of ND2, ND3, ND6, ND4L (P_P_-b module), ND4 (P_D_-a) and ND5 (P_D_-b) (early-mid stage assembly and mid-late stage assembly). The membrane arm comes together with the Q module (Q-P module) prior to the addition of the pre-assembled N module and remaining subunits (last stage of assembly)^5,9,13^.

To date, pathogenic variants have been reported in all 7 mtDNA core structural subunits (ND1–6 and ND4L), 21 of the nuclear-encoded subunits and 10 of the known CI assembly factors (reviewed in refs^14,15^). Further to this genetic heterogeneity, CI deficiency also exhibits considerable clinical heterogeneity with the spectrum ranging from severe presentations including Leigh Syndrome (LS) and fatal infantile mitochondrial disease in early childhood through to Leber Hereditary Optic Neuropathy (LHON) and exercise-induced muscle weakness which develop during young adult life.

The diagnosis of mitochondrial disease often uses a multidisciplinary approach including the histopathological and biochemical assessment of tissue biopsy samples, usually skeletal muscle, as well as molecular genetic testing. Current histochemical methods to investigate mitochondrial disease are available for some OXPHOS components (e.g. the sequential assessment of cytochrome *c* oxidase (Complex IV, COX) and succinate dehydrogenase (Complex II, SDH)) but not all; no reliable histochemical assay is available to assess CI activity. Whilst validated biochemical assays can determine the activity of individual respiratory chain enzyme complexes, for example, CI by the spectrometric assay of rotenone-sensitive NADH oxidation, consensus diagnostic protocols are not widely adopted. Furthermore, most diagnostic laboratories request at least 50 mg of muscle tissue to reliably measure enzyme activities, which represents a considerable proportion of the total amount of patient material available, particularly in children. Additionally, the biochemical assay is known to only measure the redox activity of the peripheral arm of CI (containing the N-module and Q- module). Therefore, patients with mutations residing in the membrane arm (P-module) - where only the proton pumping ability is affected rather than electron transport - may show a ‘normal’ enzyme profile following biochemical assessment despite an underlying CI defect, although this may reflect the level of mtDNA heteroplasmy in muscle tissue^8^. A confirmed diagnosis of isolated CI deficiency can facilitate appropriate molecular genetic testing to elucidate the underlying genetic cause, with screening of the mitochondrial genome preceding high throughput analysis of Mendelian candidates either by targeted gene panels, whole exome sequencing (WES) or whole genome sequencing (WGS)^16^.

Given the vast clinical and genetic heterogeneity associated with CI deficiency, accurate diagnosis is essential. A recently developed quadruple immunofluorescent assay to assess mitochondrial respiratory chain defects through the immunodetection of NDUFB8 (a CI subunit), COX-1 (a Complex IV subunit), porin (a mitochondrial mass marker) and laminin (a myofibre membrane marker) has been shown to provide an objective and reliable quantitative method for the assessment of CI and CIV protein abundance relative to the mitochondrial mass in individual muscle fibres within a single transversely-orientated, 10 µm muscle section^17^. We have extended this original study to assess CI status in skeletal muscle biopsies from 25 patients with proven pathogenic variants in proteins leading to a biochemical defect of isolated CI activity, including nuclear-encoded CI structural subunits, CI assembly factors, or one of the 7 mtDNA-encoded CI structural subunits. We demonstrate that the IHC assay has clear diagnostic potential, particularly for patients with Mendelian-inherited defects and propose that the IHC assay should form part of the multidisciplinary approach for the diagnostic investigation of patients with suspected CI deficiency.

## Results

### Mitochondrial Respiratory Chain (MRC) profiles

We assessed 25 skeletal muscle biopsies taken from patients with genetically-confirmed pathogenic variants either shown or predicted to cause isolated CI deficiency to validate a recently-developed immunofluorescence assay (IHC) within a diagnostic setting. The clinical, biochemical and molecular genetic characteristics of this patient cohort are shown in Table 1. The IHC assay, detecting CI subunit NDUFB8, CIV subunit COX-1 and porin (a marker of mitochondrial mass), was performed in all muscle sections (Supplementary Figs S1, S2 and S3), and fibres were classified according to Z scores – where any fibres with a Z score under -3SD were classified as deficient. The IHC results (NDUFB8 immunoreactivity), presented as a percentage of fibres deficient in CI – calculated by totalling the percentage of fibres classified as negative, intermediate negative and intermediate positive fibres (Table 2) - were subsequently correlated with the diagnostic biochemical findings, presented as residual CI activity (Table 1).

**Table 1:** Clinical, biochemical and molecular genetic characteristics of our patient cohort with isolated Complex I deficiency.

Patient	Gender	Adult/Paediatric	Clinical Presentation	Gene	Genetic Defect	Residual Complex I activity	Mutation Load
**Nuclear-encoded Complex I structural subunits**							
P1^a^	F	Paediatric	IUGR and oligohydramnios, FTT, mild hypertrophic cardiomyopathy	*NDUFB3*	Homozygous c.64 T > C, p.(Trp22Arg)	33%	n.a.
P2^b^	F	Paediatric	IUGR. Acute life-threatening event, age 20 days, required intubation. Hypertrophic cardiomyopathy	*NDUFB3*	Homozygous c.64 T > C, p.(Trp22Arg)	32%	n.a.
P3^c^	F	Paediatric	Oligohydramnios. IUGR. Poor feeding at birth. MRI brain and echocardiogram normal. Age-appropriate skills. Family history of previous neonatal death	*NDUFB3*	Homozygous c.64 T > C, p.(Trp22Arg)	35%	n.a.
P4	F	Paediatric	Leigh syndrome	*NDUFS4*	Compound heterozygous c.99-1 G > A + c.416_417delinsC, p.(Glu139Alafs*50)	39%	n.a.
P5	F	Paediatric	Consanguineous, first cousin parents; Leigh-like syndrome; elevated lactates	*NDUFS4*	Homozygous exon 3 and 4 deletion	37%	n.a.
P6	M	Paediatric	Infantile-onset mitochondrial disease; marked lactic acidosis	*NDUFS6*	Homozygous c.316_319delGAAA, p.(Glu106Glnfs*41)	5%	n.a.
P7	F	Paediatric	Leigh syndrome	*NDUFS2*	Homozygous c.998 G > A, p.(Arg333Gln)	42%	n.a.
P8	F	Paediatric	Leigh-like syndrome; elevated serum lactates	*NDUFS3*	Homozygous c.642_644delTGA, p.(Asp214del)	26%	n.a.
**Nuclear-encoded Complex I assembly factors**							
P9	F	Paediatric	Leigh-like syndrome; elevated lactates	*NDUFAF6*	Compound heterozygous c.805 C > T, p.(His269Tyr) and c.581-7 A > G, p.(Leu193_Gly194insValIle)	26%	n.a.
P10	F	Paediatric	Lethal infantile mitochondrial disease presentation; presented day 1 with persistent lactic acidosis; died at 9 weeks	*NDUFAF6*	Homozygous c.659 C > A, p.(Thr220Lys)	45%	n.a.
P11	F	Paediatric	Presented at 8 months; developmental regression, rotatory nystagmus bilaterally; elevated blood and CSF lactate; extensive basal ganglia and brainstem changes on MRI	*NDUFAF5*	Compound heterozygous c.826 C > T, p.(Arg276*) and c.848 C > T, p.(Ala283Val)	44%	n.a.
P12	M	Paediatric	Myoclonic seizures, developmental delay	*FOXRED1*	Compound heterozygous c.612_615dup, p.(Ala206Serfs*15) and c.1261 G > A, p.(Val421Met)	31%	n.a.
P13	M	Paediatric	Hypertrophic cardiomyopathy at birth; severe metabolic acidosis (18–30 mmol/L); died at 2 days of age	*ACAD9*	Compound heterozygous c.868 G > A, p.(Gly290Arg) and c.976 G > C, p.(Ala326Pro)	13%	n.a.
P14	M	Adult	Exercise intolerance, muscle cramps, elevated serum lactate	*ACAD9*	Compound heterozygous c.1150 G > A, p.(Val384Met) and c.1168 G > A, p.(Ala390Thr)	13%	n.a.
P15^d^	M	Adult	Exercise intolerance, unable to perform sustained aerobic exercise; normal strength; normal ECG and echocardiogram; normal resting lactate, normal CK	*TMEM126B*	Homozygous c.635 G > T, p.(Gly212Val)	36%	n.a.
**Mitochondrial DNA-encoded Complex I structural subunits**							
P16^e^	F	Adult	Exercise intolerance, persistent lactic acidaemia	*MTND1*	m.3356 T > C, p.(Met17Thr)	3%	92%
P17	M	Paediatric	Leigh syndrome	*MTND3*	m.10158 T > C, p.(Ser34Pro)	44%	90%
P18	M	Paediatric	Leigh syndrome	*MTND3*	m.10197 G > A, p.(Ala47Thr)	n.d.	93%
P19	M	Paediatric	Leigh syndrome	*MTND5*	m.13514 A > G, p.(Asp393Gly)	27%	66%
P20^f^	F	Paediatric	Chronic renal failure, myopathy and persistent lactic acidosis	*MTND5*	m.12425delA, p.(Asn30Thrfs*7)	16%	85%
P21	F	Paediatric	Bilateral ptosis, ophthalmoplegia, pyramidal tract signs, elevated blood and CSF lactates	*MTND5*	m.13094 T > C, p.(Val253Ala)	59%	58%
P22	M	Adult	Mitochondrial myopathy, elevated lactates	*MTND5*	m.13513 G > A, p.(Asp393Asn)	39%	60%
P23	M	Paediatric	Leigh syndrome	*MTND5*	m.13513 G > A, p.(Asp393Asn)	38%	77%
P24	F	Adult	Elevated CK, muscle pain and fatigue, myopathy	*MTND5*	m.13513 G > A, p.(Asp393Asn)	100%	45%
P25	M	Paediatric	Failure to strive, myopathy, increased brainstem signal on MRI, lactic acidosis	*MTND5*	m.13513 G > A, p.(Asp393Asn)	100%	63%

Residual Complex I activities, normalised to the activity of the matrix marker enzyme citrate synthase, are expresses as a percentage of mean control values.

Residual Complex I activity and mtDNA mutation load measured in muscle. Key: IUGR, intrauterine growth restriction; FTT, failure to thrive; ECG, electrocardiogram; CK, creatinine kinase; ^a,b,c,d,e^published patients: ^a^P1 = Patient 3 in Alston *et al*.^16^, ^b^P2 = Patient 2 in Alston *et al*.^16^, ^c^P3 = Patient 6 in Alston *et al*.^16^, ^d^P15 = Patient S1 in Alston *et al*.^20^, ^e^P16 = Patient 1 in Gorman *et al*.^27^, ^e^P20 = Patient published in Alston *et al*.^26^, F; Female, M; Male, n.a.; not applicable, n.d.; not determined.

**Table 2:** Percentage of Complex I (CI) deficient fibres detected with the IHC assay.

	Patient ID	IHC findings - % of CI deficient fibres	Total number of fibres analysed
**Nuclear-encoded Complex I structural subunits**	P1	79%	4372
	P2	93%	559
	P3	39%	13422
	P4	98%	5964
	P5	89%	4683
	P6	79%	880
	P7	99%	5337
	P8	96%	7154
**Nuclear-encoded Complex I assembly factors**	P9	99%	9504
	P10	96%	5355
	P11	26%	7352
	P12	96%	1708
	P13	100%	2684
	P14	100%	239
	P15	100%	131
**Mitochondrial DNA-encoded Complex I structural core subunits**	P16	92%	696
	P17	90%	5842
	P18	0%	7302
	P19	0%	2427
	P20	100%	3795
	P21	0%	1311
	P22	0%	3341
	P23	0%	2730
	P24	0%	675
	P25	0%	785

### Group 1: Nuclear-encoded CI structural subunits

**Figure 1:**
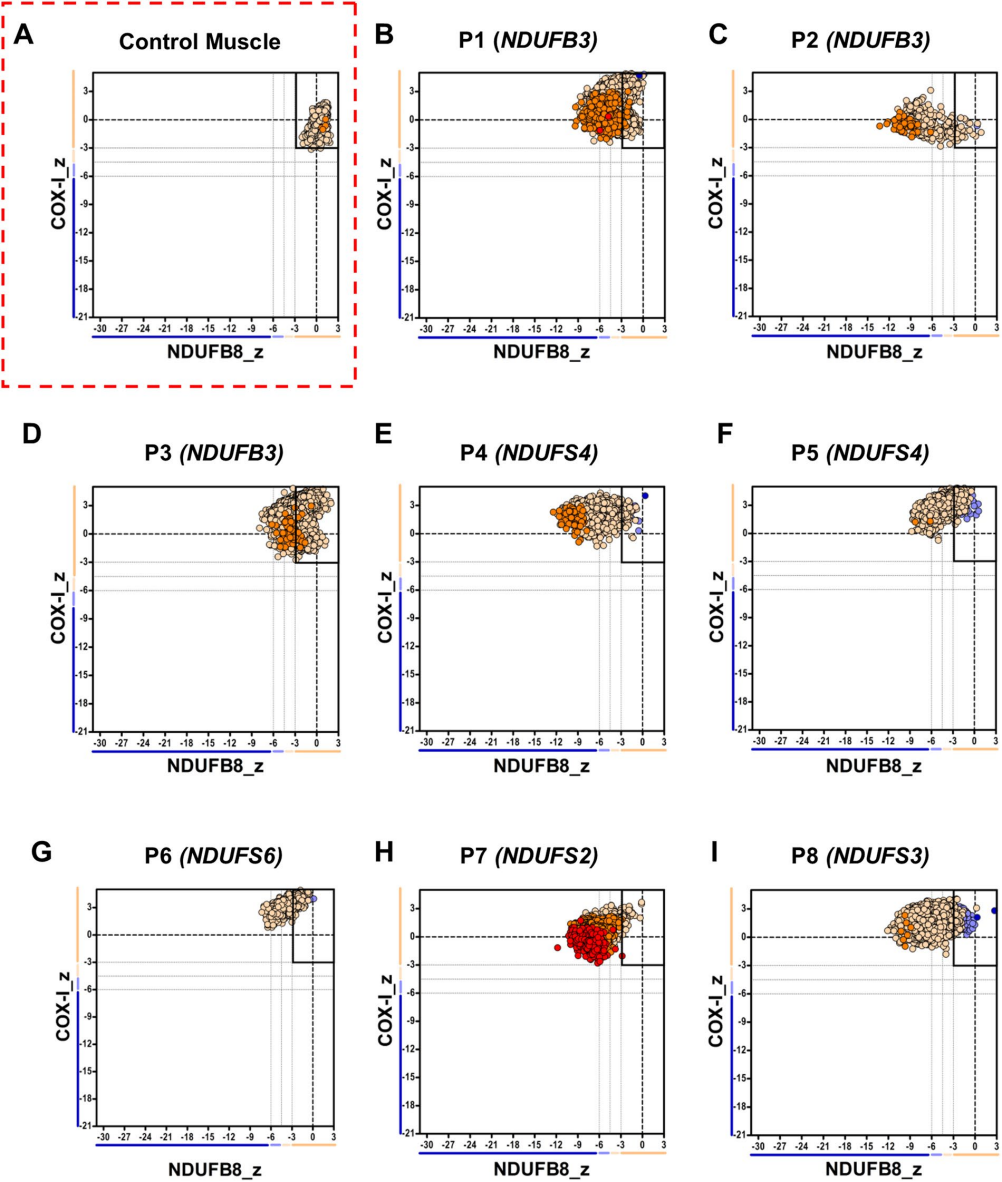
Mitochondrial respiratory chain expression profile linking complex I, complex IV and porin levels in patients with isolated Complex I deficiency caused by defects in nuclear-encoded Complex I subunits. Graphs show complex I and complex IV expression profile from (**A**) Normal adult control and patients with (**B–D**) homozygous c.64T>C, p.(Trp22Arg) *NDUFB3* variant, P1–n = 4372 fibres analysed, P2–n = 559, P3–n = 13422 (**E**) compound heterozygous *NDUFS4* variant, P4, n = 5964 (**F**) Homozygous exon 3 and 4 deletion in *NDUFS4*, P5, n = 4683 (**G**) homozygous *NDUFS6* variant, P6, n = 880 (**H**) Homozygous *NDUFS2* variant, P7, n = 5337 (**I**) Homozygous *NDUFS3* variant, P8, n = 7154. Each dot represents a single muscle fibre, colour co-ordinated according to its mitochondrial mass: very low – blue, low – light blue, normal – beige, high – orange, very high - red. Black dashed lines represent the SD limits for the classification of the fibres. Lines adjacent to X and Y axis represent the levels of NDUFB8 and COX-1: beige: normal (<−3), light beige: intermediate (+) (−3 to −4.5), light blue: intermediate (−) (−4.5 to −6) and blue: deficient (>−6). Bold dashed lines indicate the mean expression level of normal fibres.

All eight patients who have pathogenic variants in nuclear-encoded CI structural core or accessory subunits (Group 1) showed varying levels of decreased NDUFB8 immunoreactivity (representing a decrease in NDUFB8 protein abundance) when compared to control muscle (Supplementary Fig. S1). Further analysis revealed that the proportion of CI-immunodeficient fibres ranged between 39% and 99% across this group of patients. The mitochondrial respiratory chain profiles (Fig. 1), showing the NDUFB8 and COX-1 protein abundance in conjunction with mitochondrial mass in individual fibres, also illustrates this as most fibres analysed were outside of the normal range for NDUFB8 (Z-score from -3SD to 3 SD) – causing a shift in their distribution on the plot to the left - despite the normal levels of COX-1 (subunit of complex IV) in virtually all fibres assessed (n = 131–13422 fibres analysed; dependent on the size of muscle section). These findings were in agreement with the respiratory chain biochemical findings, where a decrease in residual CI activity was observed (Table 1).

### Group 2: Nuclear-encoded CI assembly factors

**Figure 2:**
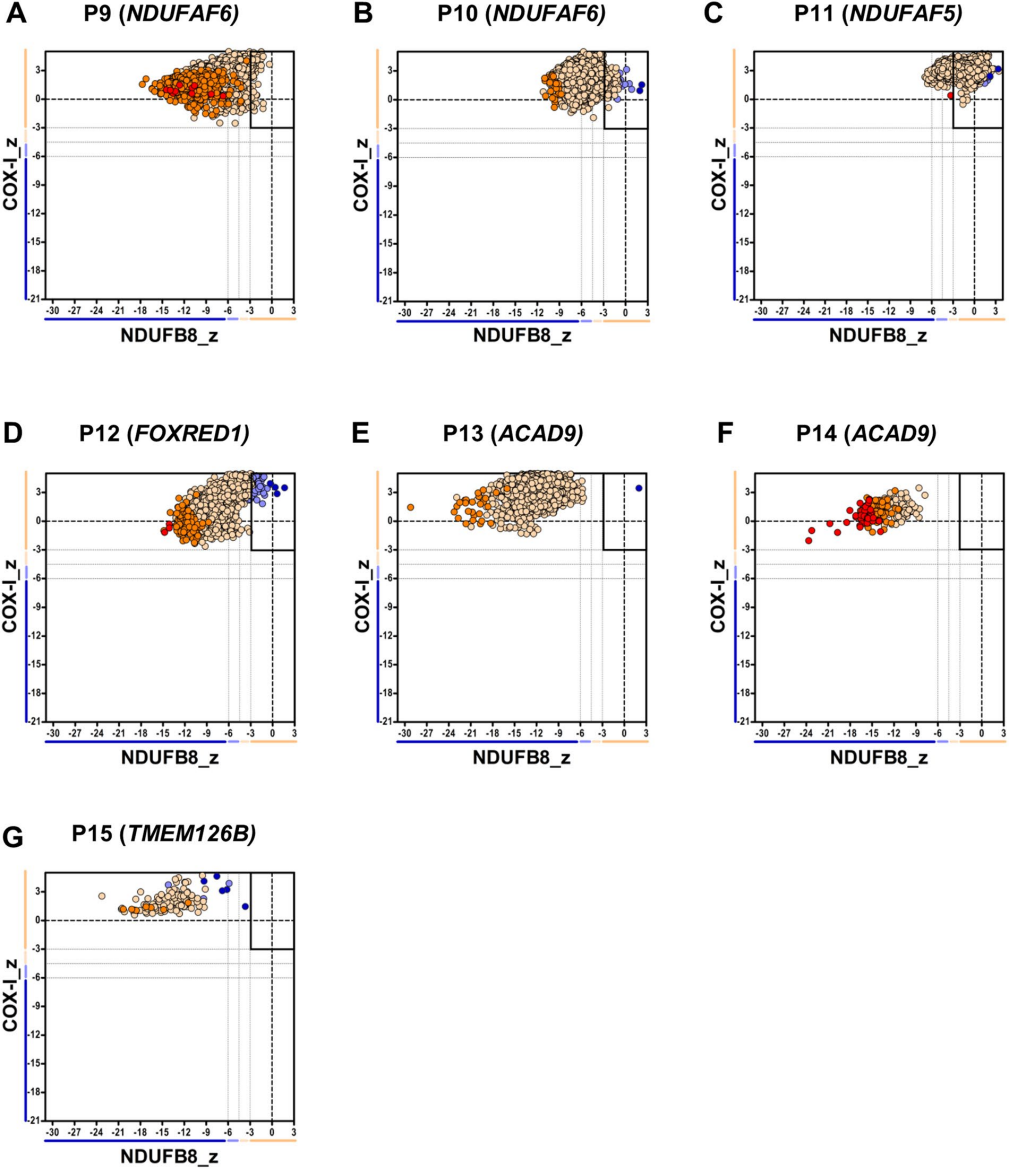
Mitochondrial respiratory chain expression profile linking complex I, complex IV and porin levels in patients with isolated Complex I deficiency caused by defects in nuclear-encoded Complex I assembly factors. Graphs show complex I and complex IV expression profile from patients with (**A**) Compound heterozygous *NDUFAF6* variant, P9, n = 9504 fibres analysed (**B**) Homozygous *NDUFAF6* variant, P10, n = 5355 (**C**) Compound heterozygous *NDUFAF5* variant, P11, n = 7352 (**D**) Compound heterozygous *FOXRED1* variant, P12, n = 1708 (**E–F**) Compound heterozygous *ACAD9* variant, (**E** = P13, n = 2684, **F** = P14, n = 239) (**G**) Homozygous *TMEM126B* variant, P15, n = 131. Each dot represents a single muscle fibre, colour co-ordinated according to its mitochondrial mass: very low – blue, low - light blue, normal – beige, high – orange, very high - red. Black dashed lines represent the SD limits for the classification of the fibres. Lines adjacent to X and Y axis represent the levels of NDUFB8 and COX-1: beige: normal (<−3), light beige: intermediate (+) (−3 to -4.5), light blue: intermediate (−) (−4.5 to −6) and blue: deficient (>−6). Bold dashed lines indicate the mean expression level of normal fibres.

Similar to patients in Group 1, all seven patients with pathogenic variants in nuclear-encoded assembly factors (Group 2), displayed a severe loss of NDUFB8 subunit immunoreactivity (Supplementary Fig. S2). With the exception of P11, further quantification showed that the percentage of CI-deficient fibres was > 96%, highlighting the severe loss of NDUFB8. Specifically, the assay detected a complete loss of NDUFB8 immunoreactivity (100% CI-deficient fibres) in patients P13, P14 and P15 all of whom had pathogenic variants in either the *ACAD9* or *TMEM126B* genes; these encode CI assembly factors involved in the biogenesis of the proximal part of the P module (P_P_). Only P11, who has compound heterozygous pathogenic variants in the *NDUFAF5* gene, encoding a CI assembly factor involved in an early step of the biogenesis of the holoenzyme, maintained a relatively normal level of NDUFB8 (26% CI-deficient fibres). As previously observed in patients from Group 1, the mitochondrial respiratory chain profiles from patients in Group 2 (Fig. 2) similarly showed a “shifting to the left”, but to a greater extent. Again, the IHC findings correlated with available biochemical results which show a more severe decrease in muscle CI activity (Table 1).

### IHC shows variable results in patients with mutations in mtDNA-encoded CI subunits (Group 3)

**Figure 3:**
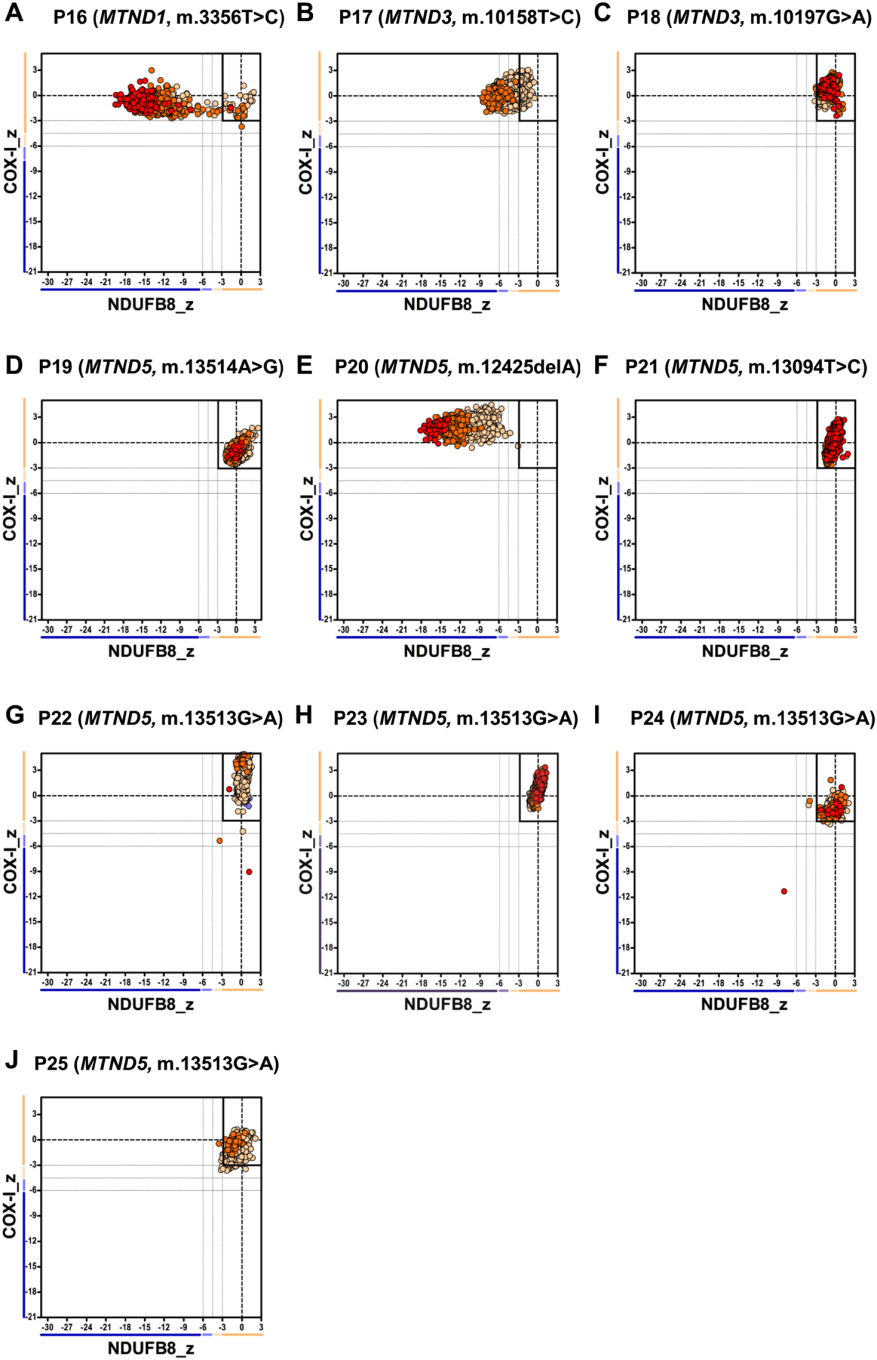
Mitochondrial respiratory chain expression profile linking complex I (NDUFB8), complex IV and porin levels in patients with isolated Complex I deficiency caused by defects in mtDNA-encoded Complex I subunits. Graphs show complex I and complex IV expression profile from patients with (**A**) m.3356T>C *MTND1* variant, P16, n = 696 fibres analysed (**B**) m.10158T>C *MTND3* variant, P17, n = 5842 (**C**) m.10197G>A *MTND3* variant, P18, n = 7302 (**D**) m.13514A>G *MTND5* variant, P19, n = 2427 (**E**) m.12425delA *MTND5* variant, P20, n = 3795 (**F**) m.13094T>C *MTND5* variant, P21, n = 1311 (**G**) m.13513G>A *MTND5* variant, P22, n = 3341 (**H**) m.13513G>A *MTND5* variant, P23, n = 2730 (**I**) m.13513G>A *MTND5* variant, P24, n = 675 (**J**) m.13513G>A *MTND5* variant, P25, n = 785. Each dot represents a single muscle fibre, colour co-ordinated according to its mitochondrial mass: very low – blue, low - light blue, normal – beige, high – orange, very high - red. Black dashed lines represent the SD limits for the classification of the fibres. Lines adjacent to X and Y axis represent the levels of NDUFB8 and COX-1: beige: normal (<−3), light beige: intermediate (+) (−3 to −4.5), light blue: intermediate (−) (−4.5 to −6) and blue: deficient (>−6). Bold dashed lines indicate the mean expression level of normal fibres.

Patients harbouring mutations in mtDNA genes encoding core structural subunits of Complex I (Group 3, n = 10) showed more heterogeneous results. The IHC assay detected 100% CI-deficient fibres, representing a complete loss of NDUFB8 immunoreactivity, in three previously-reported patients with high levels of pathogenic mtDNA variants in muscle; P16 who has a m.3356 T > C, p.(Met17Thr) *MTND1* variant, P17 who harbours a m.10158 T > C, p.(Ser34Pro) *MTND3* variant and P20 who harbours a m.12425delA *MTND5* frameshift mutation (see Supplementary Fig. S3 and Table 2). As expected, the mitochondrial respiratory chain profiles for these three patients were all shifted to the left, consistent with the loss of NDUFB8 immunoreactivity associated with preserved COX-I immunoreactivity (Fig. 3).

The remaining 7 patients (P18, P19, P21, P22, P23, P24 and P25) showed NDUFB8 immunoreactivity similar to control muscle (Supplementary Fig. S4 and Table 2). The mitochondrial respiratory chain profiles demonstrated normal respiratory chain function as all fibres fell within the normal range (with Z scores between -3SD and 3 SD) (Fig. 3). Of these 7 patients, P22- P25 all harbour a common pathogenic m.13513 G > A, p.(Asp393Asn) *MTND5* variant, P19 harbours a m.13514 A > G, p.(Asp393Gly) variant, P21 harbours a m.13094 T > C, p.(Val253Ala) variant whilst P18 has high levels of a m.10197 G > A, p.(Ala393Thr) *MTND3* variant. Detection of normal levels of immunoreactive NDUFB8 subunit in these patients could be due to these variants only affecting the catalytic function of CI rather than the assembly of the enzyme complex.

### Patients with mutations in mtDNA-encoded CI subunits and normal NDUFB8 profile also display normal NDUFS3 levels

**Figure 4:**
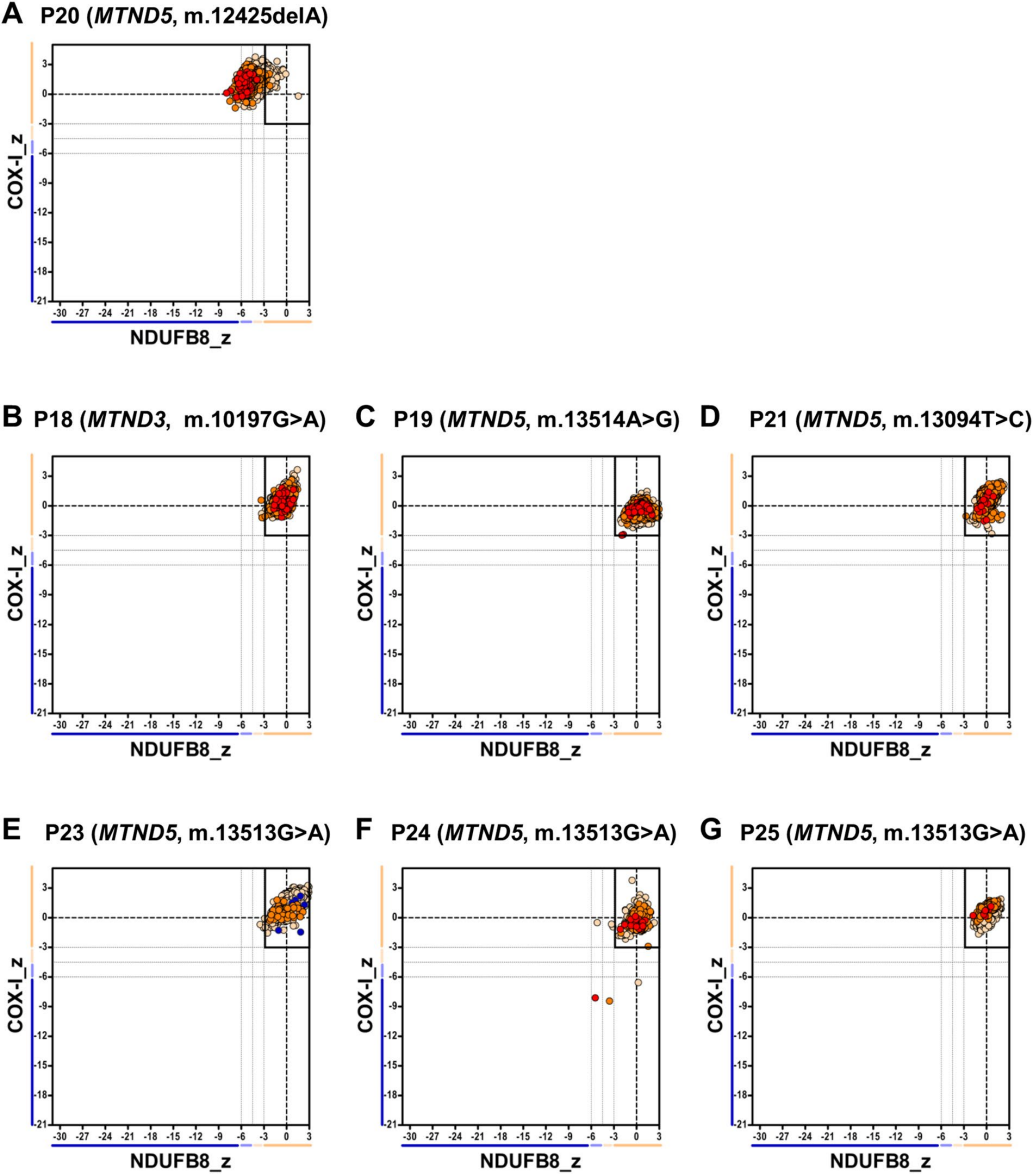
Mitochondrial respiratory chain expression profile linking complex I (NDUFS3), complex IV and porin levels in patients with Isolated complex I deficiency caused by defects in mtDNA-encoded Complex I subunits. Graphs show complex I and complex IV expression profile from patients with (**A**) m.12425delA *MTND5* variant, P20, n = 5536 fibres analysed (**B**) m.10197G>A *MTND3* variant, P18, n = 6645 (**C**) m.13514A>G *MTND5* variant, P19, n = 2730 (**D**) m.13094T>C *MTND5* variant, P21, n = 3979 (**E**) m.13513G>A *MTND5* variant, P23, n = 10009 (**F**) m.13513G>A *MTND5* variant, P24, n = 575 (**G**) m.13513G>A *MTND5* variant, P25, n = 1168. Each dot represents a single muscle fibre, colour co-ordinated according to its mitochondrial mass: very low – blue, low - light blue, normal – beige, high – orange, very high - red. Black dashed lines represent the SD limits for the classification of the fibres. Lines adjacent to X and Y axis represent the levels of NDUFB8 and COX-1: beige: normal (<−3), light beige: intermediate (+) (−3 to −4.5), light blue: intermediate (−) (−4.5 to −6) and blue: deficient (>−6). Bold dashed lines indicate the mean expression level of normal fibres.

Since interrogation of NDUFB8 immunoreactivity failed to detect CI deficiency in 7 of the 10 patients with mtDNA-encoded structural CI mutations, we further investigated whether any deficiency could be detected using an alternative, but commercially-available CI antibody targeting NDUFS3 – a subunit which is integrated during the early stage of complex I assembly. The IHC assay was performed on 10μm muscle sections from P18, P19, P20, P21, P23, P4 and P25 using antibodies against NDUFS3, porin, COX-I and laminin, and showed normal NDUFS3 immunoreactivity (Supplementary Fig. S5). As shown in Fig. 4, the mitochondrial respiratory chain profile for P20 shows a shift of fibres to the left whilst for all the remaining patients also tested with NDUFS3 fall into the normal range – these findings are the same as the assessment with the NDUFB8 antibody

### BN-PAGE assessing the steady-state levels of fully-assembled Complex I

To further interrogate the IHC results observed in the subset of patients displaying normal NDUFB8 and NDUFS3 immunoreactivity, we performed BN-PAGE analysis to assess the steady-state levels of fully assembled CI (980 kDa) where muscle was available (P18, P19, P21, P22, P23 and P25). P17 was also included as a positive control in this analysis given the IHC assay had shown down-regulated levels of NDUFB8 immunoreactivity. Samples prepared for BN-PAGE analysis retain OXPHOS complexes in their structural and active form, permitting the investigation of any effects on the assembly of the holoenzyme or catalytic activity. Since we had previously measured residual CI activity spectrophotometrically, we only assessed the levels of fully assembled complex I, using complex II assembly as a control.

**Figure 5:**
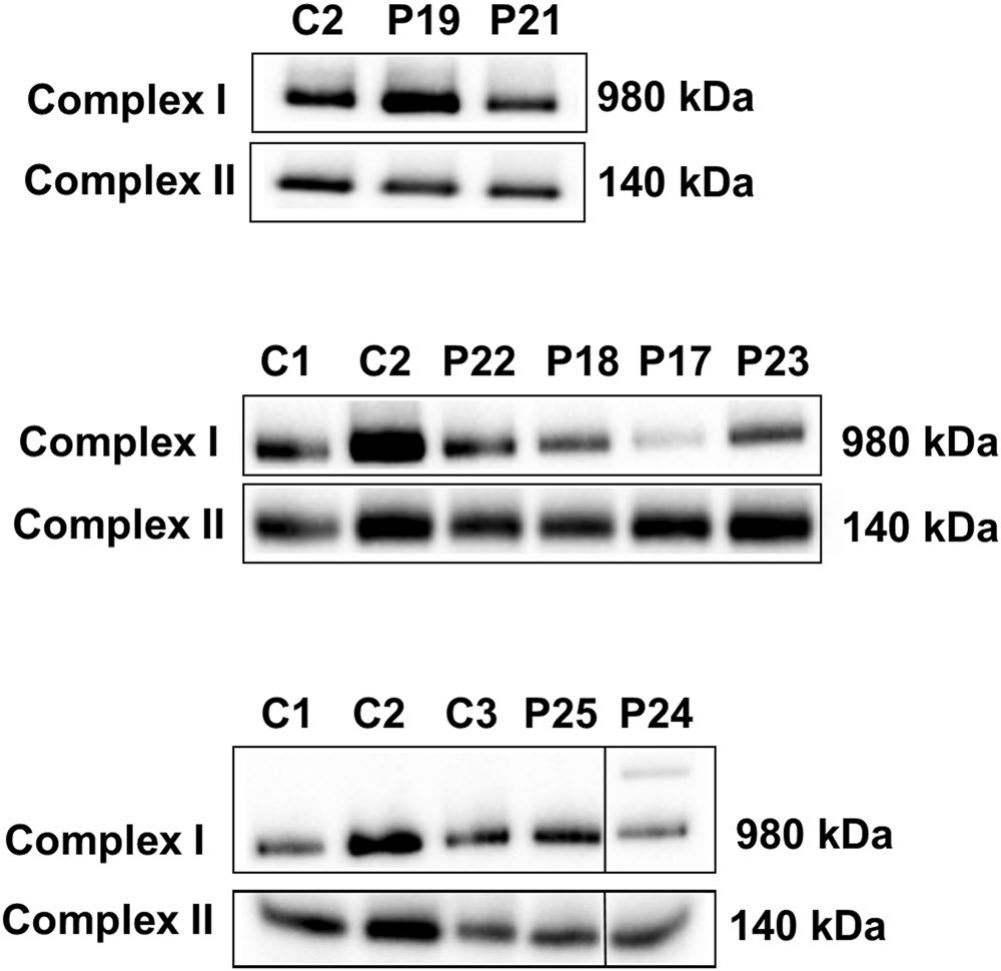
Analysis of Complex I assembly by BN-PAGE. Complex I assembly profiles were analysed using one dimensional blue native polyacrylamide gel electrophoresis (BN-PAGE) (4-16% gradient). Analysis showed a decrease in fully-assembled CI in patients P17, P18, P23 and P24, whilst normal assembly is seen in patients P19, P21, P22 and P25. Complex II was used a loading control. Both OXPHOS complexes were detected by immunoblotting using subunit specific antibodies – NDUFB8 (Complex I) and SDHA (complex II). The original, full length blots are included in the Supplementary Information File (Supplementary Fig. S6).

Analysis of muscle mitochondrial fractions by BN-PAGE revealed a decrease in steady-state levels of fully-assembled CI in P17, P18, P23 and, P24 (Fig. 5) when compared to controls, indicating that the mutations harboured by these patients affect levels of fully-assembled CI. As complex II activity is normal in these patients, we targeted SDHA subunit and used the steady-state levels of assembled complex II (detected by immunoreactivity against the SDHA subunit) as a loading control. By contrast, patients P19, P21 and P22 showed normal levels of fully assembled CI, in line with the IHC assay results, therefore likely indicating that these mutations affect the activity of the complex rather than CI assembly.

The level of CI was also normal in P25, correlating with the findings from the IHC (Fig. 5). Furthermore, the residual biochemical CI activity was also 100%, indicative that the m.13513 G > A, p.(Asp393Asn) variant in this case has no major effect on the activity or assembly of CI despite the relatively high level of mtDNA heteroplasmy (Table 1).

## Discussion

This study aimed to assess and validate the application of a recently developed quadruple immunofluorescent OXPHOS assay to the diagnosis of patients with isolated mitochondrial CI deficiency. The IHC assay, which detects NDUFB8 as a marker of CI integrity, was successful in detecting CI deficiency in 18 of the 25 patients tested, including all patients with pathogenic variants in nuclear genes encoding CI structural subunits and assembly factors. However, only 3 of the 10 patients harbouring pathogenic mtDNA variants showed a consistent decrease in NDUFB8 immunoreactivity and thus protein abundance, later confirmed using a further commercially-available antibody against NDUFS3, another key component of CI. We chose to optimise the IHC assay using a commercially-available and widely-used antibody against NDUFB8. The subunit is assembled at the mid/late stage of assembly, alongside the mtDNA-encoded core structural subunit, ND5. Although, it would have been preferable to use an antibody targeting a mtDNA-encoded subunit, such as ND1 (interrogating the early stage of CI assembly), there are currently no robust, commercially-available antibodies raised against this or other mtDNA subunits, important to consider if the assay is to be adopted across diagnostic centres.

Using this assay, we could detect CI deficiency in patients that harboured mutations in nuclear genes encoding either the accessory, (P1, P2, P3 = NDUFB3 subunit of the P-D region of the P-module, P4 and P5 = NDUFS4 subunit of the N-module, P6 = NDUFS6 subunit of the N-Module, P7 = NDUFS2 subunit of the Q-module) or core subunits (P8 = NDUFS3 subunit of the Q-module) of CI, in line with the biochemical findings. Of interest, we note that despite patients 1, 2 and 3 harbouring the same genetic variant (p.Trp22Arg in *NDUFB3*) and similar residual CI activities, the IHC findings showed variable levels of NDUBF8 immunoreactivity (P1 = 79%, P2 = 93% P3 = 39%). However, these findings are consistent with the steady-state levels of the NDUFB8 subunit of CI on western and BN-PAGE originally reported by Alston and colleagues, where it was shown that P1 and P2 (P3 and P2 respectively in ref.^16^) had decreased protein levels when compared to P3 (P6 in ref.^16^). Taken together, the collective data highlight the potential of the IHC assay to be a diagnostic tool for such cases and shows that all mutations in Group 1 exerted a similar effect on steady-state levels of fully assembled CI as previously documented^16,18,19^.

Similarly, the IHC assay was able to diagnose all patients with genetic variants in CI assembly factors. As expected, mutation of these genes led to a decrease in CI assembly and accordingly our IHC assay recorded a marked loss of CI immunoreactivity in many of the fibres analysed; a complete loss of NDUFB8 immunoreactivity (100% CI-deficient fibres) was noted in P13, P14 (*ACAD9* variants) and P15 (*TMEM126B* variant), consistent with previous reports of severe CI deficiency in *ACAD9* and *TMEM126B* mutations^20–23^. These two assembly factors, alongside with NDUFAF1, ECSIT and TIMMDC1 form the ‘Mitochondrial CI assembly’ (MCIA) complex^24^ which is associated with the assembly of the P-_P_ submodule (that contains the ND2, ND3, ND4-L and ND6 mitochondrial- encoded subunits). Since depletion of TMEM126B leads to the accumulation of the Q/P_P_-a (Q module-ND1) intermediate, it seems that NDUFB8 is unstable and as such degraded before the complete assembly of the holoenzyme, explaining why a complete loss of NDUFB8 protein is observed in all fibres^24,25^.

For patients with pathogenic variants in mtDNA-encoded CI structural subunits, the IHC assay was able to detect CI deficiency in 3 patients (P16, P17 and P20). The m.12425delA *MTND5* mutation in P20 leads to a truncated form of the ND5 subunit which has previously been shown to have a severe impact on CI assembly^26^. This was supported by our IHC results where we observed a complete loss of NDUFB8 immunoreactivity (100% CI-deficient fibres). Similarly, P16 harbours a m.3356 T > C, p.(Met17Thr) *MTND1* variant at high levels in muscle associated with a severe defect in CI assembly^27^, corroborating our IHC results ( > 90% CI-deficient fibres). Finally, P17 harbours a m.10158 T > C, p.(Ser34Pro) *MTND3* variant, leading to a marked decrease in NDUFB8 immunoreactivity and steady-state levels of fully-assembled CI; together these results show that the IHC assay confidently detects the deficiency associated with mtDNA variants which severely impact upon CI assembly.

The remaining 7 patients, where normal NDUFB8 (and, where tested, NDUFS3) immunoreactivity was detected, harbour well-characterised pathogenic variants in either the *MTND5* (P19, P21, P22, P23, P24 and P25) or *MTND3* genes (P18). It has been shown that missense variants in these proteins predominantly affect CI redox activity whereas mutations in the *MTND1*, *MTND2* and *MTND6* genes have a more severe impact on complex assembly^28–33^. We speculate that the patient mutations in which we found normal levels of CI based on both IHC and BN-PAGE analysis (P19: *MTND5*, m.13514 A > G, p.(Asp393Gly); P21: *MTND5*, m.13094 T > C, p.(Val253Thr); P22: *MTND5*, m.13513 G > A, p.(Asp393Asn)) are likely to only affect the catalytic site of the holoenzyme without disturbing CI stability and assembly. In line with this, the m.13514 A > G *MTND5* mutation (P19) has been shown to affect the redox activity of CI^34–36^. The change of amino acid at position 393 (which is part of a putative quinone-reactive site of the enzyme), causes a loss of the putative quinone-reactive site, thereby leading to a decline in CI activity^37,38^ and validating the IHC findings. Patients harbouring mtDNA variants which only affect CI catalytic activity are likely to show normal IHC profiles.

Furthermore, many studies have also shown that defects in the *MTND3* and *MTND5* genes display variable biochemical phenotypes; either decreased redox activities as determined by biochemical spectrophotometric assays, decreased levels of fully assembled CI or both^18,31,34–36,39–45^. This supports the variability found in our results as the m.13513 G > A, p.(Asp393Asn) variant, the most frequently occurring mutation in the *MTND5* gene^43^, can manifest differing effects: either a decrease in fully assembled CI (P23 and P24) or normal levels of assembled CI (P25)^18,34,35,38–43^. Somewhat surprisingly, P25 (mean m.13513 G > A heteroplasmy level of 63% in muscle) also presented with normal redox activity and CI assembly profiles, likely due to the confounding factor of mutant load. While most pathogenic mtDNA variants cause a biochemical defect only when the levels of mutations mtDNA exceeds 80–90%^46^, lower thresholds are reported in patients with CI deficiency due to structural subunit mutation^35,41^. In the case of P25, it could be possible that the pathogenic threshold in this patient is higher, thereby explaining the absence of a biochemical defect despite the presence of relatively high mutation levels and an associated clinical phenotype.

Finally, we detected normal NDUFB8 immunoreactivity but decreased levels of fully-assembled CI in patient P18 harbouring a pathogenic m.10197 G > A, p.(Ala47Thr) *MTND3* variant. This has previously been associated with decreased redox activity and diminished levels of fully-assembled CI^31,44,45^; unfortunately, due to inadequate amounts of skeletal muscle tissue from this patient, we were unable to verify if an effect on activity was also present. Our IHC data indicate, however, that the subunit must be present as the NDUFB8 level was normal. Interrogating NDUFS3 levels also showed normal results, suggesting that our IHC assay is less able to identify CI defects associated with pathogenic variants causing milder defects in CI assembly.

We believe our assay contributes to available diagnostic tools for studying mitochondrial diseases which are typified by extensive biochemical, genetic and clinical heterogeneity. Whilst all patients in our cohort with Mendelian CI defects showed a decrease or complete loss of NDUFB8 immunoreactivity and redox activity, patients with mtDNA variants affecting CI subunits displayed variable biochemical phenotypes. Consequently, we emphasise the importance of complete mitochondrial genome sequencing in the diagnostic work up of patients with suspected mitochondrial disease, to identify possible pathogenic variants associated with clinical symptoms that may result from mutation of mtDNA-encoded structural CI subunits.

In conclusion, our study has accessed and validated the use of a quadruple IHC assay in a diagnostic setting for identifying patients with suspected CI deficiency using a single 10 µm transverse skeletal muscle section. We have been able to directly detect varying levels of NDUFB8 protein abundance at a single cell level, a key advantage of the IHC assay in comparison to current histochemical and histological methodologies. Given these data, we have been able to provide evidence that this assay has clear diagnostic potential for patients with CI deficiency, particularly for those with mutations affecting nuclear-encoded genes, which account for ~75–80% of genetic causes of CI deficiency. Whilst the assay is not sufficiently sensitive to identify a biochemical defect associated with some very well-characterised mtDNA – or rare catalytic defects affecting either mtDNA-encoded or nuclear-encoded CI subunits – we believe that a combination of the quadruple IHC assay, in tandem with full mitochondrial genome sequencing and standard biochemical assays, can be used to investigate likely genetic causes of CI deficiency in patients with mitochondrial disease, especially when muscle biopsy sample sizes are small necessitating the analysis of cryosectioned material.

## Methods

### Tissue Samples and patient cohort

25 skeletal muscle biopsies (quadriceps muscle) of patients -both paediatrics (<16 years of age) and adults (>16 years of age), who have been investigated for mitochondrial disease and shown to have genetically-confirmed pathogenic variants attributed to isolated CI deficiency were included in this study (see Table 1 for detailed information). Measurements of the enzymatic activities of respiratory chain complexes were undertaken at one of two Mitochondrial Diagnostic laboratories, the NHS Highly Specialised Services located within the Wellcome Centre for Mitochondrial Research at Newcastle University or the Neurometabolic Laboratory at University College London Hospitals (UCLH). Muscle biopsy referral, enzyme measurements and genetic studies were all undertaken as part of the diagnostic work-up of these patients for suspected mitochondrial disease. All samples were obtained and used with informed consent. This study was approved and performed under the ethical guidelines issued by the Newcastle and North Tyneside Local Research Ethics Committees (reference 09/H0906/75) and complied with the Declaration of Helsinki. Control muscle was obtained from patients who were undergoing anterior cruciate ligament (ACL) operations and shown to have normal respiratory enzyme activities.

### Quadruple Immunohistochemistry

Quadruple immunohistochemistry was undertaken on 10 µm sections as described by Rocha *et al*.^17^. Briefly, sections were incubated with the primary antibodies (Supplementary Table S1) overnight at 4 °C followed by a three wash steps for 10 minutes. This was followed by incubation with secondary antibodies for 2 hours in 4 °C and a further incubation step with streptavidin conjugated with Alexa 647 for 2 hours at 4 °C (Supplementary Table S1). Sections were then mounted using ProLong gold antifade reagent. No-primary antibody control (NPC) sections (incubated only with the anti-laminin primary antibody), were processed alongside each muscle sample.

### Image Acquisition

Tiled fluorescent images were taken at x20 magnification using a Zen 2011 (blue edition) software and Zeiss Axio imager MI microscope, equipped with a motorised stage, an AxioCam digital camera and filter cubes detecting wavelengths at 488 nm, 546 nm, 647 nm and 750 nm. Exposure times were set for each channel to avoid over saturation – the same exposure times were then maintained across all cases.

### Statistical Analysis

#### Densitometry measurements

Fluorescent images were analysed using an in-house analysis software coded for by MatLab 2015a. The laminin immunofluorescence (750 channel) was used to detect fibres automatically. Any unwanted surfaces including those over background, fibres with poor morphology or folded were removed. The surfaces allowed for the measurement of mean intensity/optical density (OD) of 488 (COX-1), 546 (Porin) and 647 (NDUFB8) in each individual fibre. The same procedure was repeated for each NPC in order to determine the levels of non-specific binding

#### Data Analysis

Following image analysis, excel files containing the mean ODs for each case were analysed using an in-house web-based tool as previously described^14^, along with the statistical calculations within the manuscript Rocha and colleagues^17^. This analysis determined the Z scores for porin, COX-1 and NDUFB8 based on their expected levels, which were derived using data obtained from control muscle sections. Fibres were classified based on the SD limits into groups of NDUFB8 and COX-1 levels; >−3 = normal, −3 to −4.5 = intermediate positive, −4.5 to −6 = intermediate negative and <−6 = deficient/negative. Fibres were also classified into levels of porin according to Z scores (Z-score: “very low” (porin_Z < −3 SD), “low” (porin_Z between −3 SD and −2 SD), “normal” (porin_Z between −2 SD and +2 SD), “high” (porin_Z between +2 SD and +3 SD) and “very high” (porin_Z above +3 SD)).

### Blue Native–Polyacrylamide Gel Electrophoresis (BN-PAGE)

Mitochondrial fractions from both controls and patient muscle were prepared for Blue Native–Polyacrylamide Gel Electrophoresis (BN-PAGE) as previously described in detail by Olahova *et al*., 2015. The protein concentration of samples was determined using the Pierce BCA protein assay kit and absorption spectrophotometry measured at 562 nm. A minimum of 150ug of muscle mitochondria extracts were loaded on a native 4–16% BisTris gel (Life technologies) and electrophoretically separated in first dimension according to the NOVEX NativePAGE^TM^ Bis-Tris Gel system instructions (2 hours, 250 volts). Proteins were transferred onto a polyvinylidene fluoride (PVDF) membrane (Immobilon-P, Millipore Corporation) through wet transfer. Thereafter, the membrane was fixed in 8% acetic acid, washed and blocked with 5% milk for 1 hour at room temperature. Membrane was then subjected to standard immunoblotting analysis of OXPHOS complexes using primary and horseradish peroxidise conjugated secondary antibodies against NDUFB8 (980 kDa CI holoenzyme) and SDHA (140 kDa) (Supplementary Table S2).

## Electronic supplementary material

Supplementary Information
