# Supplementary material for: Using a quantitative quadruple immunofluorescent assay to diagnose isolated mitochondrial Complex I deficiency
Source: Sci Rep. 2017 Nov 15;7:15676. doi: 10.1038/s41598-017-14623-2 (PMC5688115; doi:10.1038/s41598-017-14623-2)
Supplement: Supplementary file 1 — Supplementary Information [file 41598_2017_14623_MOESM1_ESM.pdf]

Using a quantitative quadruple immunofluorescent assay to diagnose isolated mitochondrial Complex I deficiency

Syeda T. Ahmed[1], Charlotte L. Alston[1,2], Sila Hopton[1,2], Langping He[1,2], Iain P. Hargreaves[3*], Gavin Falkous[1,2], Monika Oláhová[1], Robert McFarland[1], Doug M. Turnbull[1], Mariana C. Rocha[1#], Robert W. Taylor[1,2]

[1]*Wellcome Centre for Mitochondrial Research, Institute of Neuroscience, Newcastle University, Newcastle Upon Tyne, UK*

[2]*NHS Highly Specialised Mitochondrial Diagnostic Laboratory, Newcastle upon Tyne Hospitals NHS Foundation Trust, Newcastle upon Tyne, UK*

[3]*The National Hospital for Neurology and Neurosurgery, University College London Hospitals NHS Foundation Trust, London, UK*

* Present address: School of Pharmacy and Biomolecular Sciences, Liverpool John Moores University, Byrom Street, Liverpool, L3 3AF

[#] Present address: BHF Centre of Research Excellence, The James Black Centre, King's College London, University of London, 125 Coldharbour Lane, London, SE5 9NU

Main author contact: robert.taylor@ncl.ac.uk

Type of article: Article

Supplementary Figure S1.

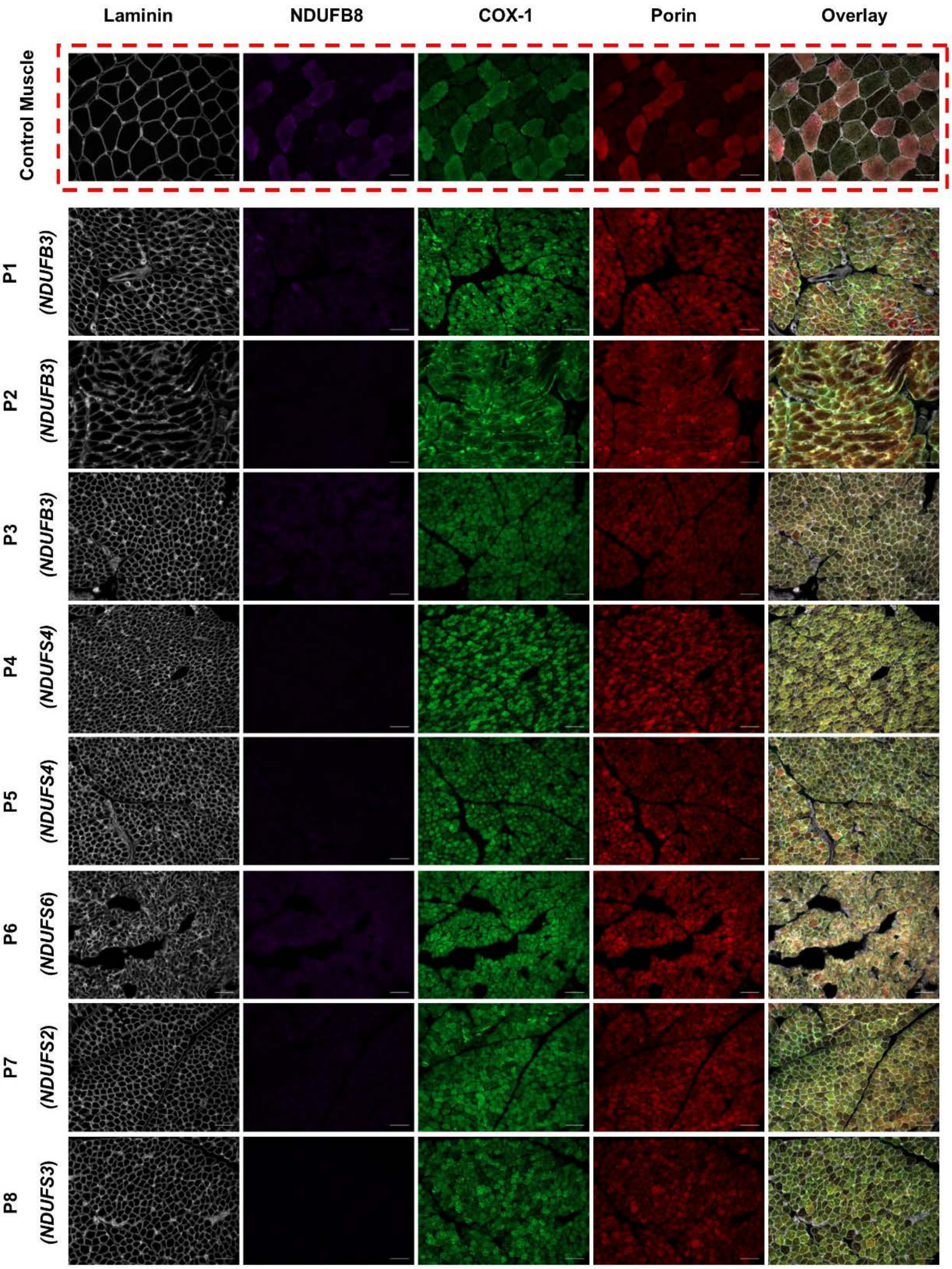

Supplementary Figure S1: Images of Complex I, IV and porin expression in skeletal muscle sections from patients with isolated Complex I deficiency caused by defects on nuclear-encoded CI subunits using the immunofluorescence assay. Fluorescent detection was used to visualise NDUFB8—purple (CI), COX –1—green (CIV), porin—red (mitochondrial mass) and laminin—white (fibre boundary marker). Control muscle (highlighted by the red box) shows normal signals for NDUFB8, COX-1 and porin. However, a marked decreased in NDUFB8 signal is seen in all patients (P1-P8) compared to the COX-1 and porin staining. Exposure times were maintained throughout all cases. Images taken at x20 magnification. Scale bars measure 50µm.

Supplementary Figure S2:

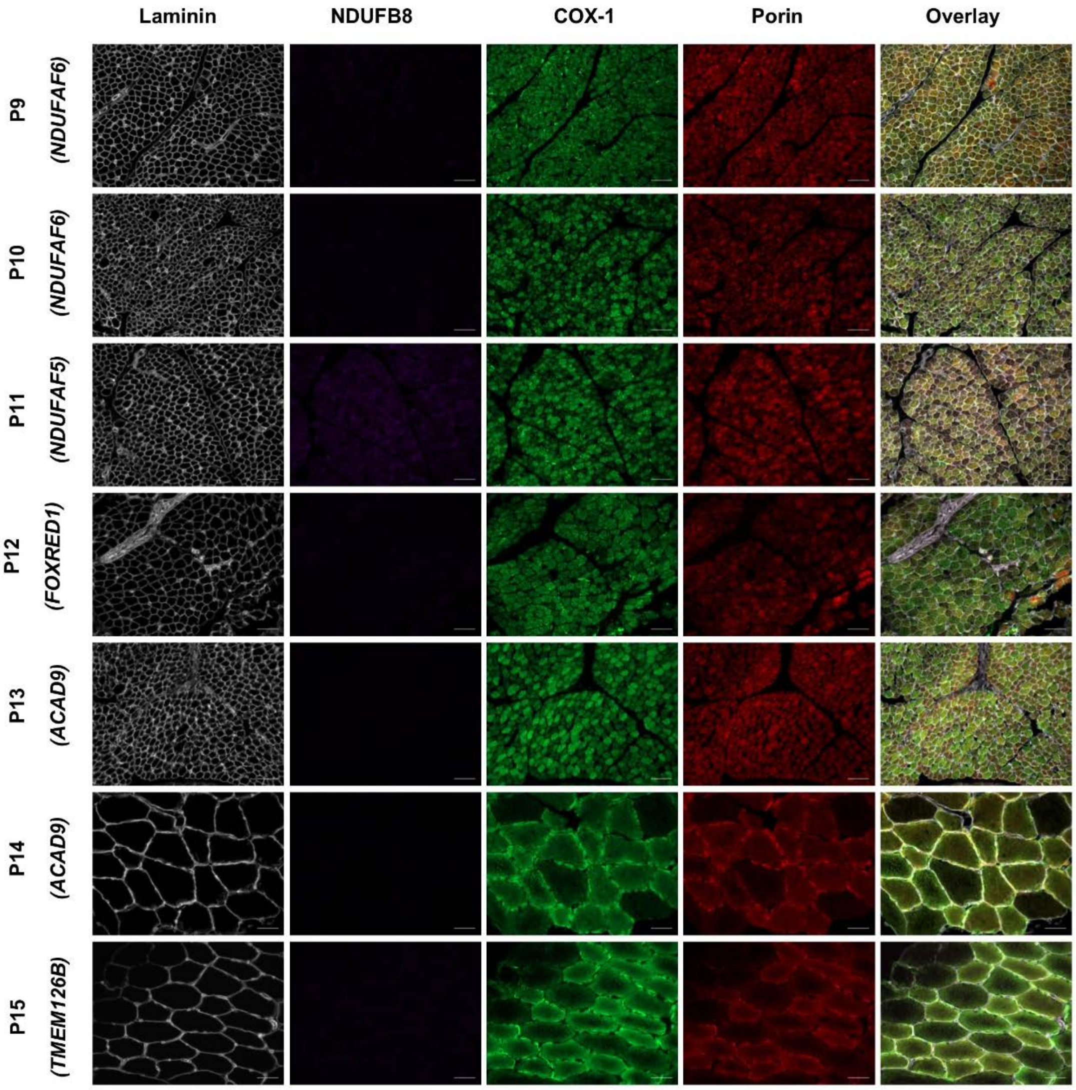

Supplementary Figure S2: Images of Complex I, IV and porin expression in skeletal muscle sections from patients with isolated Complex I deficiency caused by defects on nuclear-encoded CI assembly factors using the immunofluorescence assay. Fluorescent detection was used to visualise NDUFB8—purple (CI), COX –1—green (CIV), porin—red (mitochondrial mass) and laminin—white (fibre boundary marker). A marked decreased in NDUFB8 signal is seen in all patients (P9-P15) compared to the COX-1 and porin staining. Exposure times were maintained throughout all cases. Images taken at x20 magnification. Scale bars measure 50µm.

Supplementary Figure S3.

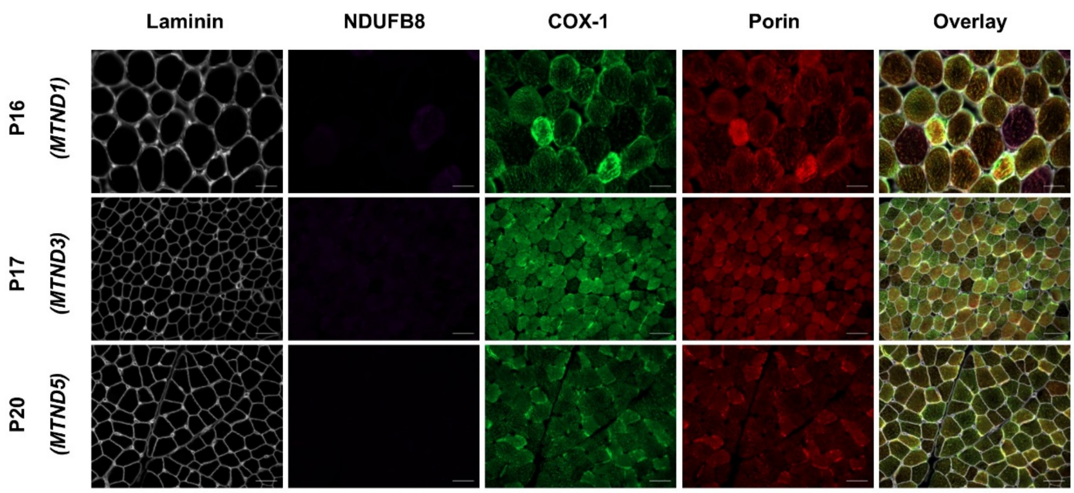

Supplementary Figure S3: Images of Complex I, IV and porin expression in skeletal muscle sections from patients with isolated CI deficiency caused by defects on mtDNA-encoded CI subunits using the immunofluorescence assay. Fluorescent detection was used to visualise NDUFB8—purple (CI), COX –1—green (CIV), porin—red (mitochondrial mass) and laminin—white (fibre boundary marker). A marked decreased in NDUFB8 immunoreactivity is seen in 3 of the 10 assessed patients in group 3; patients P16, P17 and P20 compared to the COX-1 and porin staining. Exposure times were maintained throughout all cases. Images taken at x20 magnification. Scale bars measure 50µm.

Supplementary Figure S4.

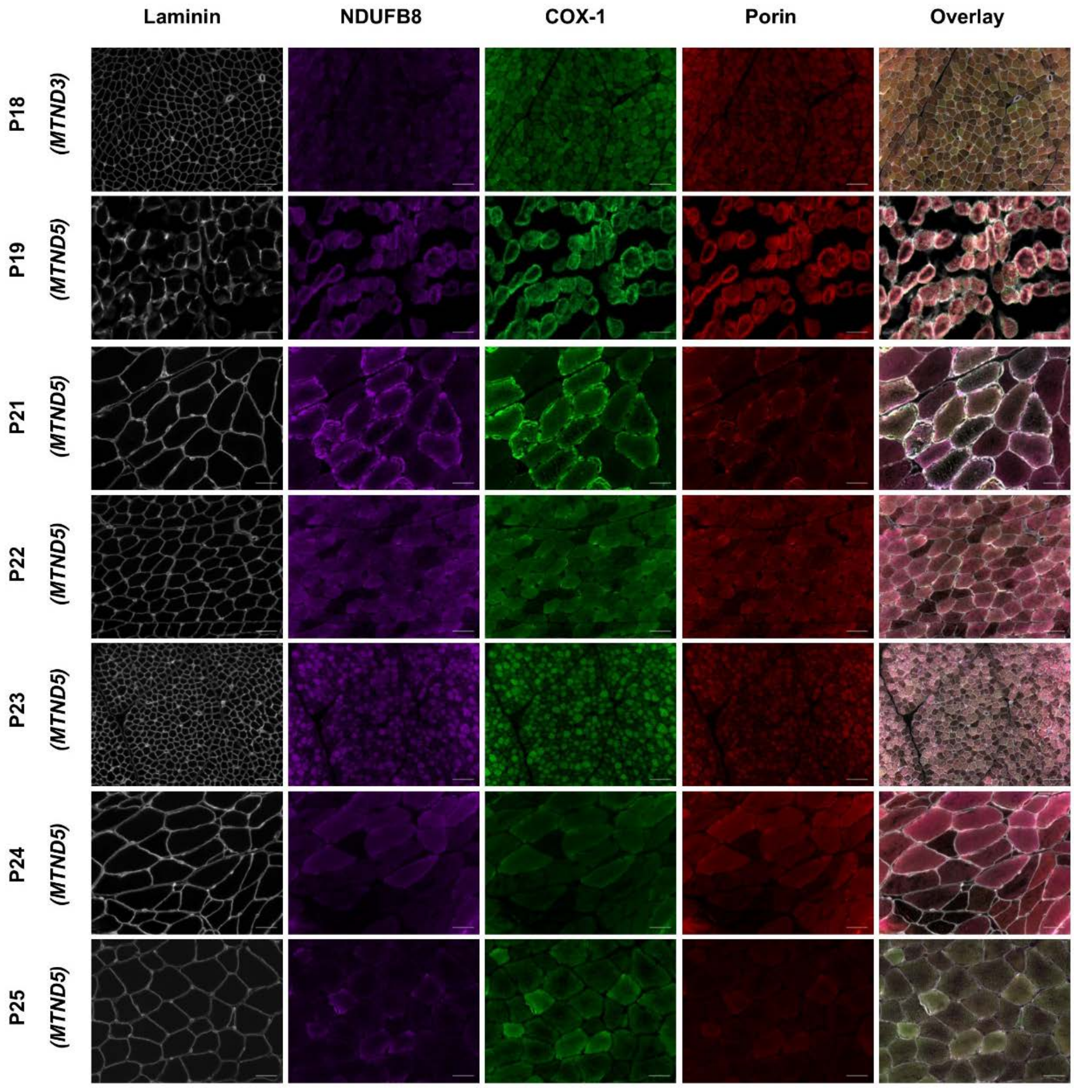

Supplementary Figure S4: Images of Complex I, IV and porin expression in skeletal muscle sections from patients with isolated Complex I deficiency caused by defects on mtDNA-encoded CI subunits using the immunofluorescence assay. Fluorescent detection was used to visualise NDUFB8—purple (CI), COX –1—green (CIV), porin—red (mitochondrial mass) and laminin—white (fibre boundary marker). In all patients, normal levels of NDUFB8 immunoreactivity is seen. Exposure times were maintained throughout all cases. Images taken at x20 magnification. Scale bars measure

Supplementary Figure S5.

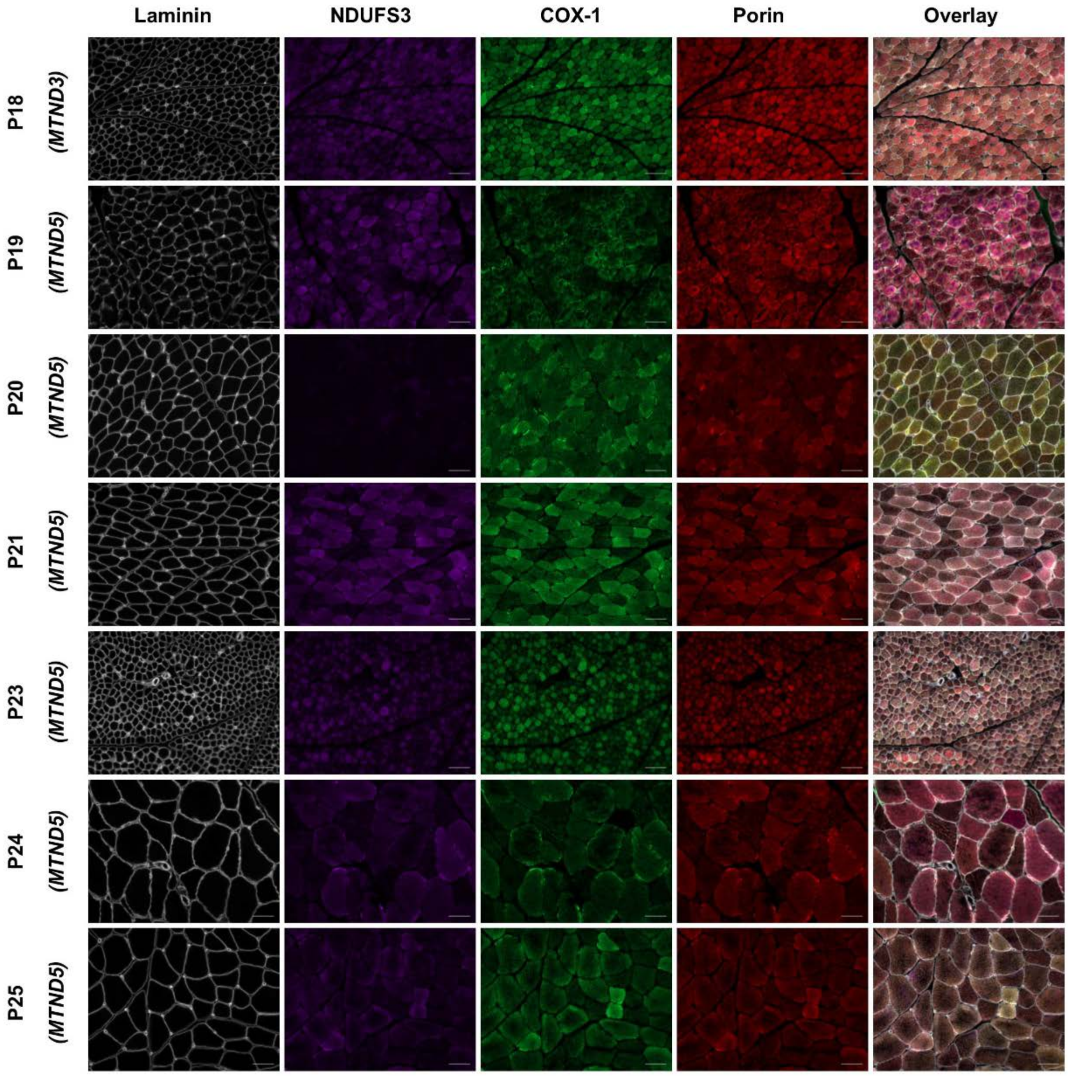

Supplementary Figure S6:

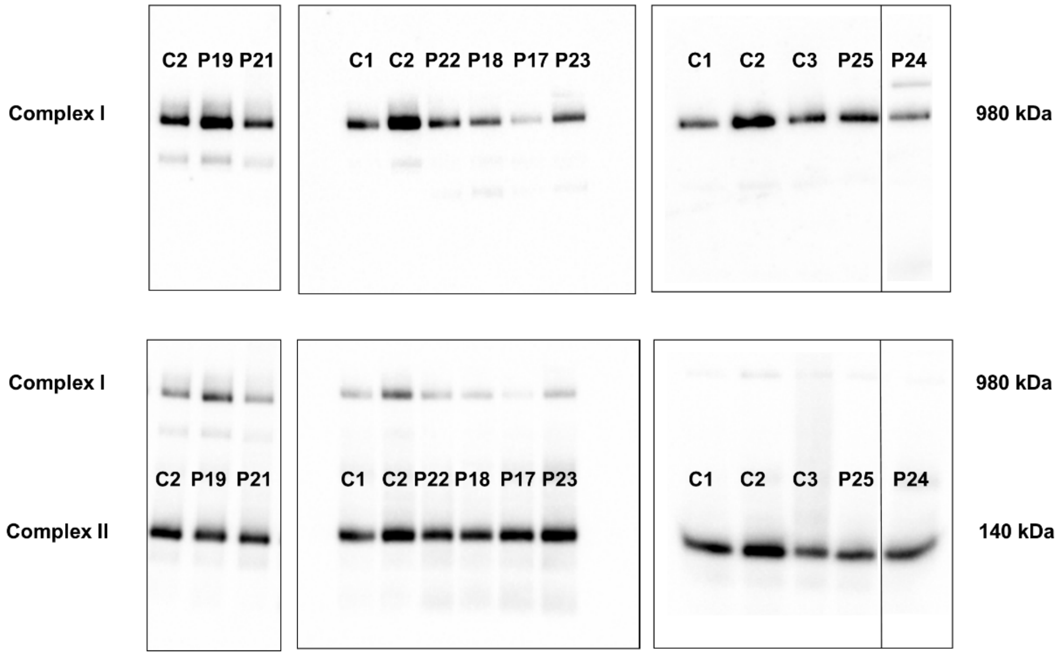

Supplementary Figure S6: Assessment of Complex I assembly. Original full length blue native polyacrylamide gel electrophoresis (BN-PAGE) (4-16% gradient) blots showing Complex I assembly profiles. Analysis showed a decrease in fully assembled CI in patients P17, P18, P23 and P24, whilst normal assembly is seen in patients P19, P21, P22 and P25. Complex II was used a loading control. Both OXPHOS complexes were detected by immunoblotting using subunit specific antibodies – NDUFB8 (Complex I) and SDHA (complex II).

Antibody	Host	Isotype	Dilution	Company (Catalogue Number)
**Primary antibodies**				
NDUFB8	Mouse	IgG1	1:100	Abcam (Ab110242)
NDUSFS3	Mouse	IgG1	1:100	Abcam (Ab110246)
MTCO1	Mouse	IGg2a	1:100	Abcam (Ab14705)
VDAC1 (porin)	Mouse	IgG2b	1:100	Abcam (Ab14734)
Laminin	Rabbit	Polyclonal (IgG)	1:50	Sigma-Aldrich (L9393)
**Secondary Antibodies**				
Anti- Mouse IgG2a Alexa Fluor 488 nm	Goat	IgG2a	1:200	Life technologies (A21131)
Anti- Mouse IgG2b Alexa Fluor 546 nm	Goat	IgG2b	1:200	Life technologies (A21143)
Anti- Mouse IgG1 Biotin	Goat	IgG1	1:200	Life technologies (n-10519)
Anti-Rabbit IgG Alexa Fluor 750 nm	Goat	Polyclonal (IgG)	1:100	Life technologies (A21039)

Supplementary Table S1: Primary and secondary antibodies used in the quadruple Immunofluorescence assay

Antibody	Host	Isotype	Dilution	Company and Catalogue Number
**Primary antibodies**				
NDUFB8	Mouse	IgG1	1:1000	Life technologies (459210)
SDHA	Mouse	IgG1	1:1000	Life technologies (459200)
**Secondary Antibodies**				
Rabbit anti- mouse	Rabbit	Polyclonal (IgG)	1:2000	Dako (P02260)

Supplementary Table S2: Primary and secondary antibodies used for BN-PAGE analysis
